# Insight into the Intermolecular Interaction and Free Radical Polymerizability of Methacrylates in Supercritical Carbon Dioxide

**DOI:** 10.3390/polym12010078

**Published:** 2020-01-02

**Authors:** Rui-Qing Li, Ming-Xi Wang, Qi-Yu Zhang, Jian-Gang Chen, Kuan Wang, Xiao-Yong Zhang, Shukun Shen, Zhao-Tie Liu, Zhong-Wen Liu, Jinqiang Jiang

**Affiliations:** 1 Key Laboratory of Applied Surface and Colloid Chemistry, Ministry of Education and School of Chemistry & Chemical Engineering, Shaanxi Normal University, Xi’an 710119, China; liruiqing@snnu.edu.cn (R.-Q.L.); wangmingxi@snnu.edu.cn (M.-X.W.); zhangqiyu@snnu.edu.cn (Q.-Y.Z.); zhangxiaoyong@snnu.edu.cn (X.-Y.Z.); zwliu@snnu.edu.cn (Z.-W.L.); jiangjq@snnu.edu.cn (J.J.); 2 College of Chemistry and Chemical Engineering, Shaanxi University of Science & Technology, Xi’an 710021, China; wangkuan@sust.edu.cn; 3 School of Materials Science & Engineering, Shaanxi Normal University, Xi’an 710119, China; shukun_shen@snnu.edu.cn

**Keywords:** intermolecular interaction, free radical polymerization, methacrylate monomer, supercritical carbon dioxide (scCO_2_), theoretical calculation

## Abstract

High pressure in situ Fourier transfer infrared/near infrared technology (HP FTIR/NIR) along with theoretical calculation of density functional theory (DFT) method was employed. The solvation behaviors and the free radical homopolymerization of methyl methacrylate (MMA), methacrylate acid (MAA), trifluoromethyl methacrylate (MTFMA) and trifluoromethyl methacrylate acid (TFMAA) in scCO_2_ were systematically investigated. Interestingly, the previously proposed mechanism of intermolecular-interaction dynamically-induced solvation effect (IDISE) of monomer in scCO_2_ is expected to be well verified/corroborated in view that the predicted solubility order of the monomers in scCO_2_ via DFT calculation is ideally consistent with that observed via HP FTIR/NIR. It is shown that MMA and MAA can be easily polymerized, while the free radical polymerizability of MTFMA is considerably poor and TFMAA cannot be polymerized via the free radical initiators. The α trifluoromethyl group (–CF_3_) may effectively enhance the intermolecular hydrogen bonding and restrain the diffusion of the monomer in scCO_2_. More importantly, the strong electron-withdrawing inductive effect of –CF_3_ to C=C may distinctly decrease the atomic charge of the carbon atom in the methylene (=CH_2_). These two factors are believed to be predominantly responsible for the significant decline of the free radical polymerizability of MTFMA and the other alkyl 2-trifluoromethacrylates in scCO_2_.

## 1. Introduction

Owing to the unique structure and superior performances, methacrylate-based monomers and polymers have attracted much attentions [1,2,3], especially for the fluorinated ones. For example, it was reported that fluorinated methacrylate polymers have excellent surface properties, impressive optical performances [4,5] and thus are extensively applied in the fields of ice-phobic coatings, functional thin film, medical materials, optical devices/fiber and nanocomposites [6,7,8,9,10]. Among the methacrylates, alkyl 2-trifluoromethacrylate(s) is suggested to be less active since a strong inductive electron-withdrawing trifluoromethyl group (–CF_3_) is connected to the α position [11]. Thus, the study on the polymerization of such monomers is of special theoretical significance, besides the potential application prospects of the corresponding polymers. Dickey et al. claimed that 2-trifluoromethyl methacrylate (MTFMA) could be polymerized by heat in the existence of the free radical initiator like benzoyl peroxide (BPO) [12]. However, no structural detail of the obtained polymer was provided. Other researchers attempted to repeat the claim and further explored the homopolymerization of trifluoromethacrylate(s) via radical initiators but most of them failed [11]. Ito et al. reported that MTFMA can be anionically homopolymerized and free radical copolymerized with methyl methacrylate (MMA) but cannot be homopolymerized via radical initiation due to the low electron density on the double bond [11]. So far there are no consistent results or explanations on the free radical polymerizability of alkyl 2-trifluoromethacrylates, which seriously restrict the research and application of such category of monomers. Moreover, the successful free radical homopolymerization of such monomers is rarely reported. The mechanistic understanding of the free radical polymerizability of such monomers is highly needed.

Recently, supercritical carbon dioxide (scCO_2_) is increasingly accepted as the most promising medium for the synthesis of fluorinated polymers because of its distinct advantages over traditional solvents. Besides its high diffusivity, low viscosity, easy accessibility of critical conditions and continuously tunable physical properties, the superiority of the inertness to effectively restraint the chain transfer reactions as well as the high solvability for many fluorinated monomers/polymers [13,14,15,16], is particularly suitable for the polymerization of fluorinated monomers. A variety of fluorinated monomers have been successfully polymerized in scCO_2_ till now [17,18,19], however, in which the alkyl 2-trifluoromethacrylates were not included. We found that the solvation behaviors of the fluorinated monomer/polymer in scCO_2_ are dominated by the interactions among the polymerization system [20]. It is also reported that the solubility of fluorinated polymers in scCO_2_ is much bigger than that of non-fluorinated ones partly due to the special attraction between the F atoms (C–F bond) and CO_2_. Moreover, the fluorine repulsion and the resulted decrease in the strength of the polymer-polymer interactions is believed to play a key role to the enhanced solubility of the fluorinated polymers in scCO_2_ [13,20]. While based on the experimental results and the theoretical calculations in perfluorooctanoic acid (FOA) + scCO_2_ system, it is reported that the interaction between the carbonyl group and CO_2_ may contribute more than that between C-F and CO_2_ to the attraction with CO_2_ [21]. So far high pressure in situ Fourier transform infrared/near infrared (HP FTIR/NIR) has been proved to be one of the most effective techniques to investigate the intermolecular interactions as well as the thermodynamic and kinetic properties in the scCO_2_ system [20,22]. Besides, ab initio/theoretical calculation is believed to be a most promising complement method to understand the interactions in scCO_2_ system [23,24,25]. Therefore, the interactions between alkyl 2-trifluoromethacrylate and CO_2_ should be revealed in order that the solvation behaviors may be better understood and the polymerization of such monomers in scCO_2_ may be successfully performed. 

**Scheme 1:**
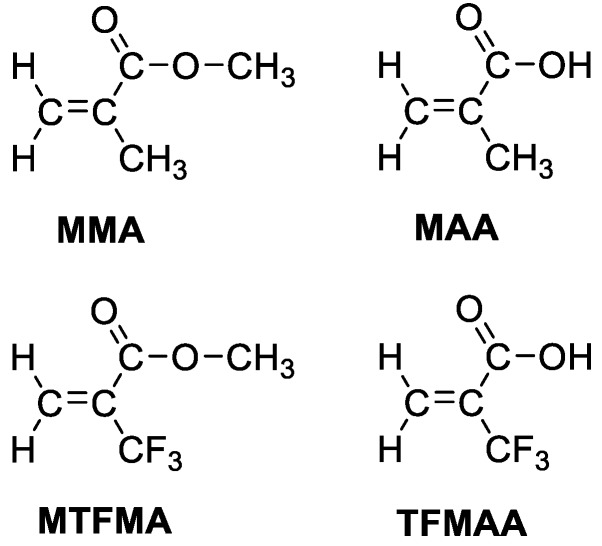
Structure of model monomers used.

In the present work, a set of patented HP FTIR/NIR system was intentionally employed [26,27], via which the solvation behaviors as well as the free radical homopolymerization of MTFMA, MAA, MMA and TFMAA in scCO_2_ were in situ monitored. Besides the HP FTIR/NIR technology, the theoretical calculation of DFT method was also used so as to discern the intermolecular interactions in monomer + CO_2_ system, to reveal the homopolymerization activity of the monomers in scCO_2_ and to understand the special contribution of –CF_3_ to the free radical polymerizability of MTFMA in scCO_2_. The structures of the monomers were shown in Scheme 1.

## 2. Experimental Section

### 2.1. Materials

2,2,2-Trifluoromethyl methacrylate acid (TFMAA, 98%, obtained from Xi’an Modern Chemistry Research Institute, China) and 2,2,2-Trifluoromethyl methacrylate (MTFMA, 97%, synthesized based on the reported method [28]) were purified by passing through a neutral alumina column twice to eliminate the residual inhibitor. Methyl methacrylate (MMA) and methacrylate acid (MAA) obtained as analytical purity, were distilled under vacuum, then passing through a neutral alumina column prior to use. Benzoyl peroxide (BPO, 97%) and azobis(isobutyronitrile) (AIBN, 98%) were recrystallized twice from methanol, dried under vacuum at 20 °C and stored at 0 °C prior to use. CO_2_ (99.999%) and N_2_ (99.99%) were used as received.

### 2.2. Apparatus

High pressure in situ FTIR/NIR experiments were performed using a PerkinElmer Spectrum 400 FTIR/NIR spectrometer supported by Spectrum Software (V 6.3.5, PerkinElmer, Bucks, UK) for data acquisition and processing. The spectrometer was intentionally modified either by replacing the standard sample accessories with a set of specially designed fiber sensor in attenuated total reflection (ATR) mode as was described in our previous work [21,29] or by integrating with a 50.0 mL stainless steel view cell to construct a high-pressure trans FTNIR in situ monitoring system [22]. The view cell was equipped with two sapphire windows, facilitating the in-line FTNIR spectroscopic monitoring of the phase behaviors during the polymerization process. ATR-FTIR spectra were recorded over a wavenumber range of 4000–600 cm^−1^ for the solvation behaviors of the methacrylate monomers in scCO_2_. The transmission FTNIR spectra were recorded over a wavenumber range of 7000–5500 cm^−1^ for the polymerization of every methacrylate in scCO_2_. Twenty scans were taken in every FTIR/FTNIR spectrum with a resolution of 2 cm^−1^.

### 2.3. Solvation Process and Phase Behaviors of the Monomers in CO_2_

The solvation process of monomers in gaseous and supercritical CO_2_ was monitored by using the high-pressure in situ ATR-FTIR monitoring system, following the similar procedure in our previous work [21,29]. The view cell was heated to the target temperature (60 °C) and then purged with N_2_ and degassed by a vacuum pump alternately for at least three times to eliminate the possible residuals in the system, then charged with a certain amount of the monomer (10 mL) via a special syringe and stirred at a speed of around 300 rpm. CO_2_ was introduced into the view cell in a stepwise fashion to solvate the previously added monomer. The FTIR spectra of the monomer were collected throughout the whole solvation process from 0 to 38.00 MPa at specified pressure intervals while stirring continued. At every selected pressure, the system was stirred for about five minutes until two identical FTIR spectra were obtained. The phase behaviors of the monomer + CO_2_ system were directly observed through the sapphire windows during the solvation process.

### 2.4. Free Radical Homopolymerization of the Monomers in scCO_2_

The free radical homopolymerization of the monomers in scCO_2_ was studied via the high-pressure FT-NIR monitoring system. The view cell was preheated to the target temperature (60 °C) and purged with N_2_ and degassed by a vacuum pump alternately for at least three times to eliminate the impurities in the system. Then the view cell was charged with a certain amount of the monomer (approximately 0.05 mol) by using a special syringe, stirred at a speed of around 300 rpm and filled with pressurized CO_2_ to approximately 12 MPa (which is slightly higher than the corresponding *P*_T_ of the monomer + CO_2_ system) so as to dissolve the added monomer. Then BPO (or AIBN) was added into the view cell via a set of high-pressure sample-in tube by the aid of the pressurized CO_2_ to initiate the polymerization. After the pressure was rapidly increased to 25 MPa (within 3 min), FT-NIR spectra were started to be in situ collected periodically during the polymerization processes, via which the monomer conversion was determined using the identical method recently reported [22]. When the system continuously polymerized for 8 hours or became cloudy [22,30,31] (where the baseline absorbance exceeded 3.0 [21]), the view cell was cooled and depressurized. After CO_2_ in the view cell was slowly released, the raw product was collected and characterized.

### 2.5. Characterization

Hydrogen nuclear magnetic resonance (^1^H NMR) and carbon-13 nuclear magnetic resonance (^13^C NMR) spectra were detected via an Avance 400 superconducting Fourier digital NMR instrument (400 MHz for proton, Bruker, Karlsruhe, Germany) in deuterated chloroform (CDCl_3_) at 25 °C, where tetramethylsilane (TMS) and the residual chloroform in CDCl_3_ were used as references of chemical shift. The Fourier transform infrared (FT-IR) spectra of the products were measured using a Tensor 27 FT-IR spectrometer (Bruker, Karlsruhe, Germany) in potassium bromide (KBr) disks in the wavenumber range of 4000 to 400 cm^−1^. The number-average molecular weight (*M*_n_) of the obtained non fluorinated polymers was analyzed using a Waters-Breeze gel permeation chromatography (GPC, Waters, Milford, CT, USA) where tetrahydrofuran (THF) was used as the eluent at a flow rate of 1.0 mL·min^−1^ and monodisperse polystyrene standards were applied to calibrate the relative molecular weight. The molecular weight of the poly 2,2,2-trifluoromethyl methacrylate (PMTFMA) was analyzed using a Microflex matrix-assisted laser desorption ionization time-of-flight mass spectrometry (MALDI-TOF mass, Bruker-Dalton, Bremen, Germany) followed a similar process to that in our recent work [22] except that dithranol was used as the matrix.

## 3. Computational Methods

The theoretical calculations have been performed using the Gaussian 2009 software package [32]. The geometries of monomer-(CO_2_)_n_ complexes as well as the monomer clusters are optimized using DFT-M062X/6-311G (2d, p) method which has been used in the similar system [33]. The absorption enthalpy(ΔH_abs_) between the monomer and CO_2_ or two monomer molecules was calculated and used to evaluate the solubility of the monomers in scCO_2_. In such calculation the solvent treatment is not considered similarly to that reported in monomer + scCO_2_ system [23]. Moreover, the atomic charge of every monomer and the corresponding monomer radical and the binding energy during the addition of the monomer with the initial free radical are also calculated in the present work. 

## 4. Results and Discussion

### 4.1. Characterization of the Monomers and Obtained Polymers in scCO_2_

**Figure 1:**
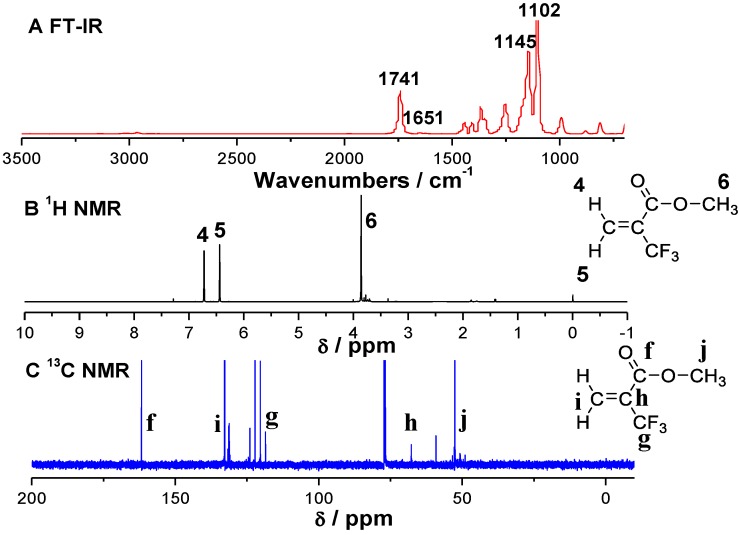
FT-IR (**A**), ^1^H NMR (**B**) and ^13^C NMR (**C**) spectra of MTFMA. FT-IR was measured in KBr. disk. ^1^H NMR spectra were recorded in CDCl_3_ at 25.0 °C, TMS and the residual chloroform in CDCl_3_ were used as references of chemical shift.

**Figure 2:**
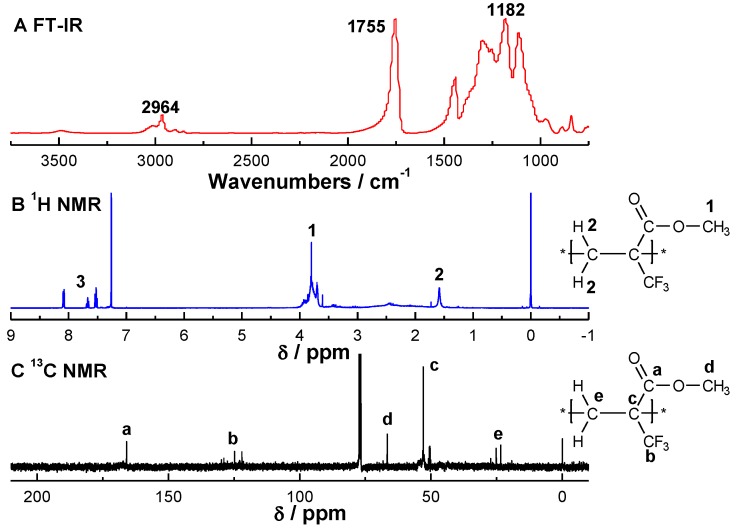
FT-IR (**A**), ^1^H NMR (**B**) and ^13^C NMR (**C**) spectra of PMTFMA synthesized in scCO_2_ at 60.0 °C under 25.0 MPa. The molar ration of MTFMA to BPO was 100:1.

**Figure 3:**
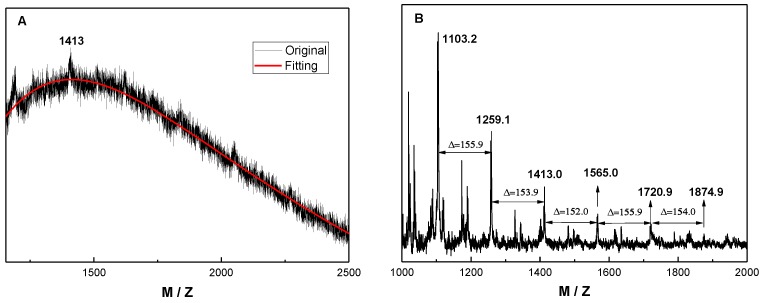
MALDI-TOF MS of PMTFMA synthesized in scCO_2_ at 60.0 °C under 25.0 MPa. (**A**) Full MS spectrum, (**B**) expansion. The matrix was dithranol.

The FT-IR (A), ^1^H NMR (B)and ^13^C NMR (C) spectra of MTFMA and the corresponding polymers (PMTFMA) are shown in Figure 1 and Figure 2, in which the attribution of every spectrum is also presented. The MALDI-TOF mass spectrum of PMTFMA is shown in Figure 3. While since the spectra of PMMA and PMAA have been reported elsewhere, such results are not listed here.

As shown in the FTIR spectrum of PMTFMA in Figure 2A, the peaks at 1114 and 1182 cm^−1^ are attributed to the stretching vibration of C-F bonds (*v*(C–F)). Moreover, as compared with FT-IR spectrum of MTFMA (shown in Figure 1A), it is seen that the peak of *v*(C=C) around 1630–1650 cm^−1^ disappeared. Additionally, *v*(C=O) is found to blue-shift from 1741 cm^−1^ (Figure 1A) to 1755 cm^−1^ due to the destruction of the π–π conjugation effect in MTFMA. As shown in the ^1^H NMR spectra of PMTFMA (Figure 2B) and MTFMA (Figure 1B), the olefinic =CH_2_ peaks in MTFMA (located at 6.44 and 6.23 ppm) completely disappeared. Furthermore, the chemical shift of –OCH_3_ slightly declined from 3.86 ppm to 3.79 ppm because of the previous p–π conjugation effect in MTFMA weakened. While the signal of the carbon atom in –CF_3_ (centered near 125 ppm) splits into multiple peaks mainly due to the spin coupling of the directly connected three F atoms, as shown in the ^13^C NMR spectra of PMTFMA in Figure 2C. More importantly, as shown in the MALDI-TOF mass spectrum of PMTFMA in Figure 3A, the molecular weight is measured as 1413 g·mol^−1^. Additionally, it is seen that the intervals of marked peaks are all close to the molecular weight of MTFMA (154.1, shown in the expansion in Figure 3B). Since the spectral results mentioned above can be well attributed and confirmed with each other, it is indicated that MTFMA has been successfully polymerized in scCO_2_.

### 4.2. Solvation Bahaviors of the Monomers in Gaseous and Supercritical CO_2_

**Figure 4:**
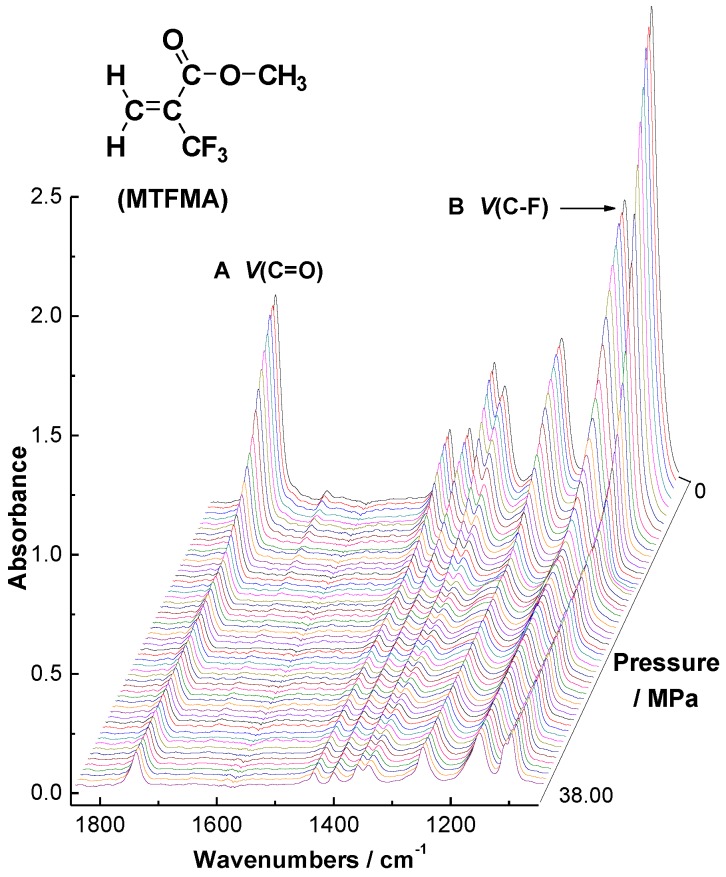
In situ FTIR spectra series of the MTFMA + CO_2_ binary system at 60.0 °C.

**Figure 5:**
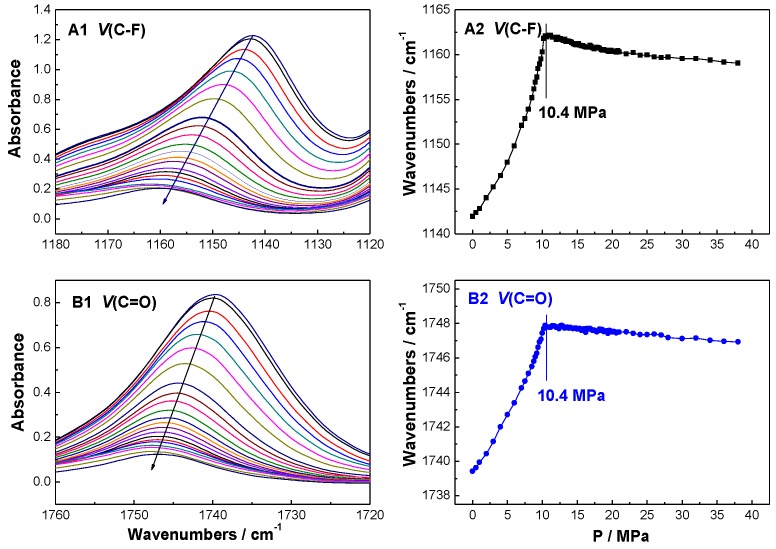
Vibrational absorption evolution of (**A1**,**A2**) *v*(C–F) and (**B1**,**B2**) *v*(C=O) in the MTFMA + CO_2_ binary system at 60.0 °C.

**Figure 6:**
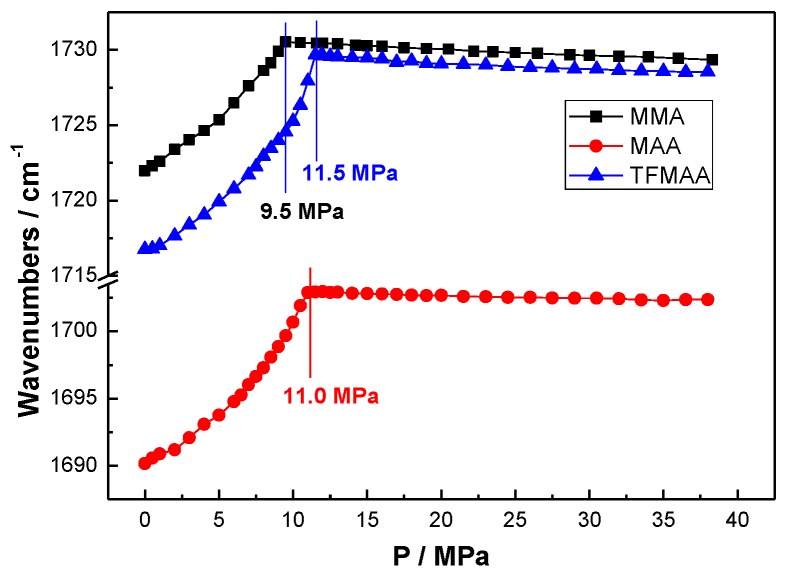
Vibrational absorption evolution of the functional groups in the MMA (MAA, TFMAA) + CO_2_ binary system at 60.0 °C.

We believe that it is the intermolecular interactions in monomer/polymer + CO_2_ system that dominate the solvation process as well as the phase behaviors of monomer/polymer in scCO_2_ and may also play an important role to the polymerizability of the monomer in scCO_2_. Previously, we presented the concept of transition pressure (*P*_T_) to demonstrate the unexpected vibrational absorption evolution of the functional groups during the solvation process of liquid monomers and polymers in gaseous and supercritical carbon dioxide [21,29]. *P*_T_ is defined as the lowest pressure at which the liquid solute could be completely dissolved or miscible with scCO_2_ under isothermal conditions. A lower *P*_T_ that the solute + CO_2_ presents, a better solubility that the solute has in scCO_2_. In the present work, the absorption band centers of C=O and C–F are extracted from the initial in situ FTIR spectra monitored via the high-pressure ATR-FTIR system and plotted versus the CO_2_ pressure. In this way, the absorption evolution of these functional groups in the MTFMA (MMA, MAA, TFMAA) + CO_2_ binary system of the probe functional groups (*v*(C–F) and *v*(C=O)) was sketched and from which the corresponding *P*_T_ of every binary system was measured, as shown in Figure 4, Figure 5 and Figure 6.

As shown in Figure 4, Figure 5 and Figure 6, it is found that the *P*_T_ of the MTFMA + CO_2_ system is measured as 10.4 MPa at 60.0 °C, either *v*(C–F) or *v*(C–O) was used as the probe. While at the identical temperature, the *P*_T_ of MMA + CO_2_, MAA + CO_2_ and TFMAA + CO_2_ system was obtained as 9.5 MPa, 11.0 MPa and 11.5 MPa, respectively. Such differences in the *P*_T_ values are attributed to the differences in the monomer structures and the resulted interactions in the corresponding monomer + CO_2_ system and are demonstrated as follows.

First, the carboxyl group and the resulted strong hydrogen bonding among the monomers are believed to negatively impact on the miscibility of the monomer with CO_2_ and thus contribute to a higher *P*_T_ of the binary system. While after the carboxyl group is esterified, the *P*_T_ is found to effectively decrease since the negative impact of hydrogen bonding is eliminated. For example, from TFMAA to MTFMA, the *P*_T_ of the corresponding binary system is found to decrease from 11.5 MPa to 10.4 MPa, from MAA to MMA, the *P*_T_ of the system is found to decrease from 11.0 MPa to 9.5 MPa.

Second, the dispersion interaction among the monomers along with the attraction between –CF_3_ of the monomer and CO_2_ plays a contrary role to the miscibility of the monomer with scCO_2_. It is inferred that the dispersion interaction may play a predominant role in solvation behaviors of most monomers/polymers in scCO_2_ [21]. In view that the dispersion interaction among the monomers increases notably with the increase of the molecular weight (*M*_n_), the corresponding *P*_T_ should also distinctly increase in the present work. While from MMA to MTFMA, the *M*_n_ increases by 54, the *P*_T_ is found to increase only by 0.9 MPa. Similarly, the identical increase of *M*_n_ from MAA to TFMAA merely results in an increment of 0.5 MPa in the *P*_T_ of the binary system. We believe that it is the attraction between CO_2_ and –CF_3_ in the fluorinated monomers, as well as the unique fluorine repulsion among the fluorinated molecules, that effectively weakens/counteracts the negative effects of the dispersion interaction among the monomers on the *P*_T_ of the binary system and thus contribute potently to the enhanced solubility of fluorinated monomers in scCO_2_.

So as to further discern the intermolecular interactions during the solvation process of the methacrylate monomers in scCO_2_, the theoretical calculations of DFT method is intentionally employed as the complement to the high pressure in situ FTIR technique.

### 4.3. Calculation of the Intermolecular Interactions in Methacrylate + CO_2_ System

**Figure 7:**
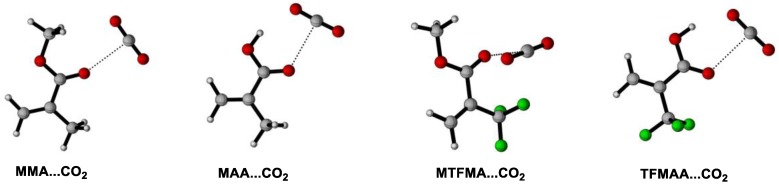
Optimized geometries of the lowest energies for M…CO_2_ clusters.

**Figure 8:**
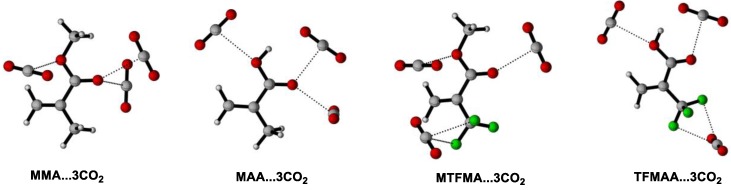
Optimized geometries of the lowest energies for M…3CO_2_ clusters.

**Figure 9:**
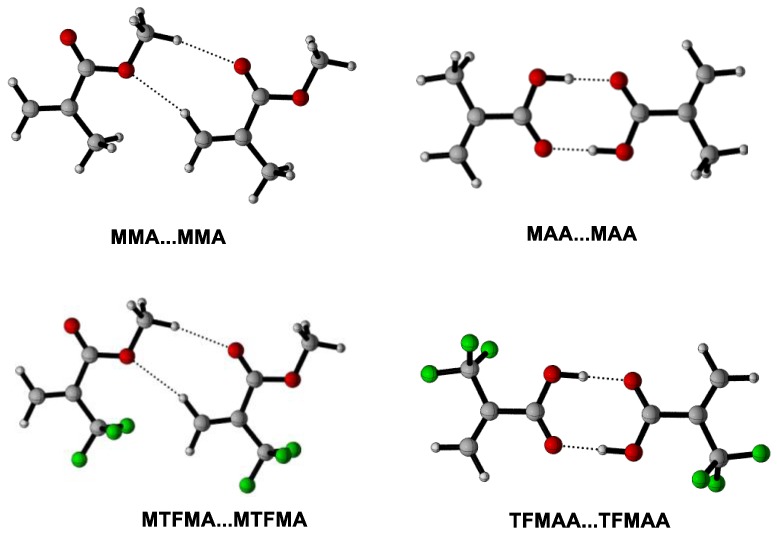
Optimized geometries of the lowest energies for M…M clusters.

We believe that the evolution of σ(A–B) (the average resultant force/interaction of the intermolecular interactions between the CO_2_ and the monomer) and σ(B-B) (the average resultant force/interaction of the self-interactions between the monomers) dominate the solvation behaviors of monomer in scCO_2_ [21,29]. So far, several function groups, such as C=O [34], C–O [35], C–F [15], are reported to definitely contribute to σ(A–B) due to the special attraction between the specific group(s) and CO_2_. It is also reported that the interaction between the carbonyl group and CO_2_ may contribute more than that between C–F and CO_2_ to the attraction of FOA with CO_2_ in FOA + scCO_2_ system [22]. In the present work, M…CO_2_ is used to represent the cluster of monomer(s) with CO_2_ under lower CO_2_ pressure and M…3CO_2_ represents the cluster under higher CO_2_ pressure. While M…M is applied to describe the cluster of pure monomers. Moreover, the absorption enthalpy(Δ*H*_abs_) (which is defined as the enthalpy change during the formation of the M…nCO_2_ cluster (n = 1, 3) or the M...M cluster) is deliberately introduced to evaluate the magnitude/intensity of σ(A–B) or σ(B–B), so as to demonstrate the evolution of the resultant force/interaction with the increase of CO_2_ pressure and to certify/corroborate the intermolecular-interaction dynamically-induced solvation mechanism in scCO_2_ system. The optimized geometries of the lowest Δ*H*_abs_ of M…nCO_2_ (n = 1, 3) and M…M, as well as the corresponding calculated Δ*H*_abs_ of M…nCO_2_ (n = 1, 3) and M…M are shown in Figure 7, Figure 8 and Figure 9 and Table 1.

It can be seen that the monomer molecule may be better solvated by CO_2_ when the pressure increases since more stable cluster of M…nCO_2_ may form (shown in Figure 7 and Figure 8). Moreover, as shown in Table 1 (Entry 1), the calculated △*H*_abs_(M…M) is found to be more than twice of △*H*_abs_(M…CO_2_), indicating that the average resultant interaction between MMA is stronger than that between CO_2_ and MMA under lower CO_2_ pressure, namely, σ(B–B) > σ(A–B). More importantly, it is clearly seen that the obtained △*H*_abs_(M…3CO_2_)(−9.2 kJ/mol) is distinctly higher than △*H*_abs_(M…CO_2_) (−3.4 kJ/mol), certifying that σ(A–B) increases with the increase of CO_2_ pressure. Contrarily, the initially strong σ(B–B) may be gradually weakened/undermined because of the slight electron redistribution along with the expansion of MMA by CO_2_ during pressuring [20]. With the increase of CO_2_ pressure, σ(A–B) may increase further, versus that σ(B–B) is increasingly weakened. There must be a pressure value where σ(A–B) may equal to σ(B–B), the macroscopic force on the MMA molecule may equilibrate and MMA may completely disperse and fully miscible with CO_2_ under isothermal condition. Such pressure is exactly the *P*_T_ of MMA + CO_2_ system, which has been accurately determined via our high-pressure FTIR monitoring system (9.5 MPa). It is inferred that, as the CO_2_ pressure further increased, σ(A–B) may probably become greater than σ(B–B), which has been partly proved and for the obtained △*H*_abs_(M…3CO_2_) the value is found to be higher than that of the initial △*H*_abs_(M…M) (−7.8 kJ/mol).

Similarly, the solvation behaviors of MTFMA in gaseous and supercritical CO_2_ can also be well understood via the calculation as well as the IDISE hypothesize. While the calculated △*H*_abs_(M…M) in MTFMA is found to be obviously higher than that in MMA (partly due to the increase in *M*_n_), resulting in a higher *P*_T_ of MTFMA + CO_2_ system.

Unexpectedly, the obtained △*H*_abs_(M…M) in MAA (−70.1 kJ/mol) or TFMAA (−103.6 kJ/mol) is found to be dramatically higher than that in MMA or MTFMA, suggesting that the σ(B–B) in these systems may be severely greater than that in MMA or MTFMA. Such strong self-interactions are believed to be mainly derived from the existence of –COOH and the resulted effective hydrogen bonding in the system. Moreover, it is clearly that the initial value of △*H*_abs_(M…M) is considerably greater than that of △*H*_abs_(M…3CO_2_) in MAA + CO_2_ system, indicating that the self-interaction of σ(B–B) may be too strong to be surpassed by σ(A–B), even CO_2_ pressure is considerably high. In this case, MAA must exist in form of clusters instead of single molecule in scCO_2_, especially in the dimer form, as shown in Figure 9. Additionally, probably owing to the strong electron-withdrawing effect of –CF_3_ in TFMAA, the intermolecular hydrogen bonding among TFMAA is predicted to be further enhanced, leading to a greater σ(B–B) in TFMAA. Such prediction has been largely proved since the calculated △*H*_abs_(M…M) in TFMAA is much greater than that of the other three methacrylate monomers, along with that the experimentally measured *P*_T_ of TFMAA + CO_2_ system is the highest among that of the four methacrylate + CO_2_ systems in the present work. It is inferred that the TFMAA…TFMAA clusters may restrain the diffusion of TFMAA in scCO_2_ and decrease the polymerizability of TFMAA in scCO_2_ to some extent.

Based on the calculation mentioned above, it is clear that the Δ*H*_abs_ may be a rational and promising function to evaluate the evolution of σ(A–B) and σ(B–B) during the solvation process of the monomers in CO_2_. In view that the predicted order of the solubility of the four monomers inscCO_2_ (MMA > MTFMA > MAA > TFMAA) is ideally consistent with that of the experimental *P*_T_ (MMA < MTFMA < MAA < TFMAA), it is believed that the previously proposed mechanism of intermolecular-interaction dynamically-induced solvation effect (IDISE) of monomer in scCO_2_ system is well certified/corroborated.

### 4.4. Free Radical Polymerization of the Monomers in scCO_2_

The free radical polymerization of the four monomers in scCO_2_ was investigated so as to understand the impacts of the monomer structure, especially –CF_3_ in the alkyl 2-trifluoromethacrylate(s), on the polymerizability of the monomer. The results are listed in Table 2.

As shown in Table 2, it is clear that MMA and MAA can be easily polymerized under the experimental conditions mentioned above. For example, after polymerized for 8 hours, the conversion of MAA reaches to 70.5% and the *M*_n_ comes up to 23,800. While for MTFMA, it is found that at the identical polymerization time to that in MAA, the conversion of MTFMA is 3.1%. Even if the polymerization lasts for 40 h, the conversion is merely 10.8%. Simultaneously, the molecular weight of the polymeric product is found to be severely low as 1634. Namely, the polymerization degree is around 10. It is shown that the free radical polymerizability of MTFMA is considerably poor, though such monomer has been polymerized via the initiation of BPO in the present work. What’s more, it is found that TFMAA cannot be polymerized by the initiation of free radical initiator in either scCO_2_ or organic solvent. Clearly, –CF_3_ may significantly decrease the free radical polymerization activity of the corresponding methacrylate(s) in scCO_2_.

We believe that the existence of –CF_3_ and the resulted strong electron-withdrawing effect may distinctly change the electron distribution in C=C. May this be the reason for the reduction of polymerizability of the MTFMA and TFMAA? Theoretical calculation of DFT-M062X/6-311G (2d, p) method was employed to understand the homopolymerization activity of the monomers in scCO_2_, especially the contribution of –CF_3_ to the free radical polymerizability of MTFMA in scCO_2_. The atomic charge of every monomer (M) and the corresponding monomer radical (IM·, produced by the addition of the monomer with the initial free radical I) and binding energy (△*E*, the energy change in the formation of IM, I + M→IM) of every monomer with I· are calculated and listed in Table 3.

As shown in Table 3 (Entry 1), it is found that after –CH_3_ is replaced by –CF_3_, the absolute value of the atomic charge of C1 in C=C distinctly decreases. For example, it declines from 0.300 e (in MMA) to 0.235 e (in MTFMA) or from 0.293 e (in MAA) to 0.223 e (in TFMAA). It is believed that such a decrease in the atomic charge of C1 in MTFMA or TFMAA is predominantly derived from the strong electron-with drawing inductive effect of –CF_3_ to C=C and the resulted electron redistribution in C=C (namely, the electron between C1 and C2 may shift to C2 to some extent) and may be mainly responsible for the decline of the free radical polymerizability of MTFMA and TFMAA. Possible reasons are presented as follows.

First, generally, C1 in C=C is much easier to be attacked by the initial free radicals since the steric hindrance of C1 is much smaller than that of C2 in methacrylate monomers along with that more favorable/stable intermediate of monomer radical IM may be produced if C1 of the monomer is bonded with the initial free radical. In this case, it is suggested that the decrease in the atomic charge of C1 may be severely unfavorable for the chain initiation. Such suggestion is believed to be verified by the calculation of the binding energy. For example, it is seen in Table (Entry 7) that the absolute value of the binding energy of MTFMA with I· (77.239 kJ·mol^−1^) is clearly found to be much lower than that of MMA with the identical I·(95.513 kJ·mol^−1^), indicating that the initiation activity of I· may decline significantly when MTFMA is used instead of MMA. Similar results can also be obtained when TFMAA is applied instead of MAA.

Second, the chain propagation activity may dramatically decline in step with the decrease of atomic charge of C1 in C=C. As shown in Table 3 (Entry 6), the absolute value of the atomic charge of C6 is found to be considerably small when MMA or MAA is in involved in the monomer radical of IM· and which is even smaller in the monomer radical of I· with MTFMA or TFMAA. Moreover, with regard to the chain propagation, the steric hindrance of C6 in IM is obviously bigger than that of C2 in the corresponding monomer, especially when MTFMA or TFMAA is polymerized. These two factors are both unfavorable for the chain growth during the free radical polymerization. In this case, the monomer should be active enough if the monomer/polymer chain is expected to continuously propagate, namely, the polymerization successfully occurs. It is inferred that the electron distribution in C=C, especially the atomic charge in C1 of the monomer, may play a key role to the free radical polymerizability of the methacrylate monomers. The bigger the absolute value of the atomic charge of C1 in C=C is, the more easily the chain propagates.

Pulsed laser polymerization (PLP) is reported to be an effective technique to determine the propagation rate coefficients (kp) of a series of monomers (MMA, MAA, styrene and vinylidene fluoride, etc.) in the free radical polymerization in both scCO_2_ and organic solvent systems [36,37,38]. While the report on such determination of the alkyl 2-trifluoromethacrylate(s) monomer (such as MTFMA or TFMAA) has not been seen/available so far. Moreover, ab initio calculations are also used to investigate the polymerization kinetics [39,40]. It is expected that these methods may be successfully applied in the next work so as to achieve further understanding on the polymerizability of alkyl 2-trifluoromethacrylate(s) as well as other fluorinated monomers in scCO_2_.

In brief, since the absolute value of the atomic charge of C1 in MTFMA or TFMAA is distinctly small as compared with that in MMA or MAA, the chain initiation as well as the chain propagation of MTFMA or TFMAA is predicted to be severely difficult to occur, which is well in accordance with what has been observed experimentally in the free radical polymerization in the present work.

## 5. Conclusions

The solvation behaviors and the free radical homopolymerization of four methacrylate monomers, including MMA, MAA, MTFMA and TFMAA in scCO_2_ were systematically investigated via HP FTIR/NIR technology along with the theoretical calculation of DFT-M062X/6-311G (2d, p) method in the present work. The intermolecular interactions of every monomer + CO_2_ system during the solvation is demonstrated and discerned via the concepts of *P*_T_, σ(A–B), σ(B–B) and Δ*H*_abs_, via which the solubility of the monomer in scCO_2_ is successfully evaluated and the previously proposed mechanism of intermolecular-interaction dynamically-induced solvation effect (IDISE) of monomer in scCO_2_ may be well verified/corroborated. More importantly, the homopolymerization activity of the four methacrylates in scCO_2_, especially the special contribution of –CF_3_ to the free radical polymerizability of MTFMA in scCO_2_ is revealed. The free radical polymerization of MMA and MAA in scCO_2_ is easy to occur, while that of MTFMA is much difficult though it does have been successfully polymerized via the initiation of BPO. TFMAA cannot be polymerized via the free radical initiators. It is believed that there are mainly two factors that are predominantly answer for the significant decline of the free radical polymerizability of MTFMA, TFMAA and other alkyl 2-trifluoromethacrylates in scCO_2_. First, the diffusion of the monomer in scCO_2_ may be restrained since the intermolecular hydrogen bonding is enhanced by –CF_3_. Second, the atomic charge of C1 in C=C may be the distinctly decreased by the strong electron-withdrawing inductive effect of –CF_3_ to C=C. Whereas there are still many issues to be further solved, the present work may be the first example that successfully combine the HP FTIR/NIR technology with the DFT method to explore and disclose the intermolecular interaction mechanism as well as the free radical polymerizability of methacrylate in scCO_2_ and is expected to improve the research and application in many related fields. 

## Figures and Tables

**Table 1:** Δ*H*_abs_ (kJ/mol) of the optimized geometries of M…nCO_2_ and M…M.

Entry	M	*M_n_* (g·mol^−1^)	*P*_T_ (MPa)	Δ*H*_abs_(M…CO_2_) M + CO_2_→M…CO_2_	Δ*H*_abs_(M…3CO_2_) M + 3CO_2_→M…3CO_2_	Δ*H*_abs_(M…M) 2M→M…M
1	MMA	100.1	9.5	−3.4	−9.2	−7.8
2	MAA	86.1	11.0	−4.4	−10.4	−70.1
3	MTFMA	154.0	10.4	−3.7	−8.2	−13.8
4	TFMAA	140.1	11.5	−4.6	−7.3	−103.6

**Table 2:** Results for the free radical polymerization of the monomers in scCO_2_^a^.

Entry	Monomer	Reaction Time/h	Conversion/% ^b^	Molecular Weight/g·mol^−1^
1	MMA	4	30.7	57817 ^c^
2	MAA	8	70.5	23800 ^c^
3	MTFMA	8	3.1	1413 ^d^
4	MTFMA	40	10.8	1634 ^d^
5	TFMAA	cannot be polymerized ^e^		

^a^ Free radical polymerization was carried out at 60.0 °C and 25.0 MPa. The molar ratio of the monomer to BPO was 100/1 (mol/mol). ^b^ Conversion was calculated via HP FT-NIR. ^c^ *M_n_* of the synthesized nonfluorinated polymers were analyzed using GPC. ^d^ *M*_n_ of the PMTFMA was analyzed using MALDI-TOF mass (as shown in Figure 2). ^e^ No polymeric product is obtained whatever the free radical polymerization of TFMAA were performed in scCO_2_, trifluorotoluene or toluene and initiated by AIBN or BPO.

**Table 3:**
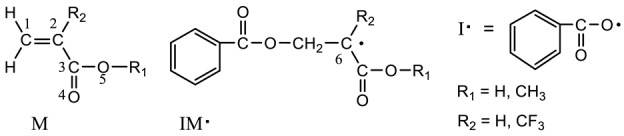
Geometric parameters and the binding energy of every monomer with I.

Entry	Parameter	MMA	MAA	MTFMA	TFMAA
	Atomic charge of R/e				
1	1C	−0.300	−0.293	−0.235	−0.223
2	2C	−0.137	−0.145	−0.245	−0.272
3	3C	0.825	0.819	0.828	0.820
4	4O	−0.619	−0.619	−0.608	−0.575
5	5O	−0.573	−0.715	−0.556	−0.685
6	6C(in IM·)	0.062	0.072	0.027	−0.009
	Binding energy/kJ·mol^−1^				
7	△E (I·+ M→IM·)	−95.513	−100.960	−77.239	−79.138
